# E-cigarette-related beliefs, behaviors, and policy support among young people in China

**DOI:** 10.18332/tid/156836

**Published:** 2023-01-23

**Authors:** Simone Pettigrew, Joseph Alvin Santos, Yuan Li, Mia Miller, Craig Anderson, Thout S. Raj, Alexandra Jones

**Affiliations:** 1 The George Institute for Global Health, University of New South Wales, Sydney, Australia; 2 The George Institute for Global Health, Beijing, China; 3 The George Institute for Global Health, Hyderabad, India

**Keywords:** e-cigarettes, vaping, young people, adolescents, policy support

## Abstract

**INTRODUCTION:**

China has recently introduced a range of e-cigarette control policies with a focus on addressing an increase in youth vaping. This study aimed to investigate a wide range of e-cigarette-related attitudes and behaviors in a national sample of Chinese adolescents and young adults to obtain insights into their exposure to, experiences with, and attitudes to e-cigarettes on the cusp of new regulations coming into force.

**METHODS:**

An online survey was administered to a sample of 1062 adolescents and young adults (aged 15–30 years) in China in November–December 2021. Quotas were applied to achieve an approximately equal gender split, representation across age sub-groups (15–17, 18–20, 21–23, 24–26, and 27–30 years), and approximately two-thirds representing low- and middle-income groups. Adjusted odds ratios (AORs) were also calculated.

**RESULTS:**

Just under half (47%) reported no knowledge of e-cigarettes and/or vaping. One in five reported ever using e-cigarettes (‘even once or twice’), 8% reported being current users, and 3% reported being daily users. Around twothirds of those who had heard of e-cigarettes/vaping had friends who vaped and had seen e-cigarette advertising. Fruit flavors were most popular, and the most frequently nominated reasons for vaping were to cut down on cigarette smoking and because a friend used them. The factors positively associated with ever use of e-cigarettes were current tobacco smoking (AOR=68.26) or previous tobacco smoking (AOR=39.15) and having friends who vape (AOR=1.76). Perceptions of addictiveness were negatively associated with ever use (AOR=0.47). Strong support was evident for most assessed e-cigarette control policies.

**CONCLUSIONS:**

The results indicate that young people in China have been able to access and use e-cigarettes, although rates of regular use are low. Generally, high levels of expressed support for a range of e-cigarette control policies among members of this age group suggest the new regulatory environment is consistent with their policy preferences.

## INTRODUCTION

China is an important context for examining e-cigarette uptake. As a country it has the largest number of smokers (around 341 million smokers representing 27% of those aged ≥15 years) and accounts for one-third of global tobacco consumption^1,2^. Given evidence that smokers are more likely to adopt e-cigarettes than non-smokers, including in China^3-5^, there appears to be vast potential for e-cigarette uptake in the population. In addition, e-cigarettes were invented in China and the country produces the majority of the world’s e-cigarette supply^6,7^, indicating potentially high levels of availability. Early in their appearance on the market, e-cigarettes were at times reported in the Chinese media to be harmless, and in some cases healthy^8^. These misperceptions may have had an ongoing effect on public perceptions of the acceptability of the devices and on likely (non)support for restrictive policies.

Another important factor is that government regulation of the e-cigarette industry in China has historically been less stringent compared to that in many other countries^9^. This prompted numerous calls for regulatory enhancements, with a primary focus on restrictions that would protect youth from e-cigarette use^5,6,9-13^. The particular focus on youth reflects research indicating that younger people in China are more likely to use e-cigarettes than older members of the population^9^, which is of concern given evidence that e-cigarettes can contain toxic chemicals and nicotine is harmful to the developing brain^14^. Dual use is especially problematic because it has been associated with higher overall nicotine intake, heightened sensitivity to smoking cues, and greater risk of cardiovascular and respiratory problems^15-17^.

New regulations have been introduced in China in 2022 that have brought e-cigarette production and marketing requirements more in line with those for tobacco cigarettes. For example, e-cigarette products destined for the domestic market now should not be sold or promoted to minors, are required to comply with specified quality control processes, must carry warnings on their packaging, can only be flavored with tobacco flavor, and cannot be sold in non-nicotine formats^13,18,19^. These policy changes have the potential to modify the trajectory of observed increases in vaping uptake among young people in recent years, although the increases have occurred from a low base^6^. Assessing young people’s e-cigarette-related attitudes and behaviors over time will be important to determine the extent to which these new policies reduce interest in and demand for e-cigarettes among members of this key consumer group.

Limited research on the factors associated with e-cigarette uptake has been undertaken in China to date. The available work has identified lower levels of awareness and usage of e-cigarettes and understanding of vaping harms compared to other countries, and high levels of variability between regions and age groups^6^. For example, the 2018 Global Adults Tobacco Survey China Project found that 49% of adults had heard of e-cigarettes^9^, while studies involving school students in Shanghai (2019) and Zhejiang (2017) found 74% and 70% awareness, respectively^4,20^. In terms of ever use of e-cigarettes, a study of university students in Hangzhou found a rate of 8%^21^, while a study of young adults in Hong Kong found a rate of 16%^3^. The motives for using e-cigarettes that have been most commonly identified in previous research are to assist with smoking cessation and because they are considered fun and fashionable^5-7^.

There is a recognized need for more detailed information about young people’s attitudes to e-cigarettes and patterns of e-cigarette use in China to inform the development of public information programs designed to address substantial knowledge deficits relating to potential harms^3,7^. Such education programs could encourage compliance with the newly introduced regulations, although little is known about the extent to which young people support the kinds of policy changes that have occurred in China and also the more comprehensive range of e-cigarette policies recommended by the World Health Organization^22,23^. Such information is important for understanding likely compliance with the more restrictive regulations that have recently come into force.

The aim of the present study was to assist in addressing these research gaps by investigating a wide range of e-cigarette-related attitudes and behaviors among Chinese adolescents and young adults to obtain insights into young people’s exposure to and experiences with e-cigarettes on the cusp of the introduction of the new suite of regulations. Attitudes towards restrictive regulations of relevance to young people were also assessed. The results provide an indication of young people’s e-cigarette-related attitudes and behaviors at a key point in time, potentially providing a comparison for subsequent work assessing the effectiveness of the regulations in generating attitudinal and behavioral change. There are important policy implications arising from the findings that can assist policymakers in China to develop and implement effective strategies to optimize young people’s compliance with the new regulatory framework.

## METHODS

As part of an international study, Pureprofile (an ISO-accredited web panel provider) was commissioned to administer an online survey to around 1000 people aged 15–30 years, in China during November–December 2021. Quotas were applied to achieve a sample characterized by an approximately equal gender split, representation across various age sub-groups (15–17, 18–20, 21–23, 24–26, and 27–30 years), and around two-thirds of respondents representing low- and middle-income groups. Individuals on Pureprofile’s data base who met age and quota eligibility were sent an invitation by email to participate in an online survey. Once they had used the link to click through to the survey, they were provided with an information sheet that informed them that: ‘This research study aims to examine e-cigarette related attitudes, intentions, and behaviors among young people’. The completion rate was high: 93% of eligible respondents clicking through to the survey provided complete data and were included in analyses (response rate data are provided in the Supplementary file Table S1). Respondents could elect to complete the survey in either Mandarin or English. The study received approval from a University Human Research Ethics Committee and all respondents provided informed consent.

The survey addressed e-cigarette-related attitudes, intentions, and behaviors, along with support for a range of e-cigarette control policy options. Survey items assessed respondent demographics (age, sex, income, education level, region of residence), use of e-cigarettes and tobacco products, attitudes to e-cigarettes^24,25^, numbers of friends and family members who vape^26^, and exposure to e-cigarette advertising^27^. To ensure comparability across product-related items, the e-cigarette and tobacco use items asked respondents to report if they ‘Never used’, ‘Previously used’, or ‘Currently use’ e-cigarettes and tobacco products ‘even just once or twice’. Those who reported being past or current e-cigarette users were asked about their motivations for vaping^26-28^, how they obtained e-cigarettes^29^, and where they used them (a new item developed for this survey). Those who reported never having used e-cigarettes were asked whether they were curious about them, had intentions to use them in the next 12 months, and would use an e-cigarette offered by a friend^26,30^. Minors (aged <18 years) were asked about their perceptions of how easy it is to access e-cigarettes^26^.

Finally, respondents were asked to indicate the extent to which they agreed with 14 e-cigarette policies that are recommended by the World Health Organization^21,22^ and/or have been examined in prior research^25,31^. The assessed policies related to restricting access to e-cigarettes, regulating product characteristics (e.g. flavors, nicotine content, and packaging), mandating product warnings, restricting advertising and banning unproven claims, and extending regulations that apply to the sale and use of tobacco products to e-cigarettes.

### Statistical analysis

Frequencies and proportions were reported for the assessed variables and chi-squared analyses were conducted to identify any significant differences according to sex, age, and tobacco use status. A significance threshold of p<0.001 was applied to account for the large number of comparisons. This was a pragmatic approach to establish a significance threshold that reduces the risk of spurious associations due to family-wise errors while allowing more robust relationships to be identified. Means and standard deviations were calculated for the policy support items.

A multivariable logistic regression model was then generated to explore factors associated with e-cigarette use, the results of which are presented as crude and adjusted odds ratios (AORs) and their 95% confidence intervals (95% CI). The dependent variable was the ‘ever use’ of e-cigarettes, for which current and previous e-cigarette use were combined into a single category and compared against never use of e-cigarettes. Independent variables were age, sex, education level (completed school, certificate/diploma, or university qualification), income (low, middle, high), region of residence (east, central, west); tobacco smoking status (never, previous, current); numbers of family members and friends who used e-cigarettes; the perceived harmfulness and addictiveness of e-cigarettes; and exposure to e-cigarette advertising. An advertising exposure variable was calculated by assessing exposure to at least one of the three forms of e-cigarette advertising measured in the survey (Internet, television/cinema/streaming services, petrol stations). The regression analysis significance threshold was p<0.05. Statistical analyses were conducted using Stata BE V17.0 for Windows (StataCorp LLC, College Station, TX).

## RESULTS

Data from 1062 respondents were included in analyses. The demographic and product use profiles of the sample are given in the Supplementary file Table S2. As per recruitment targets, there was an approximately even split between males (48%) and females (52%) and a spread across age sub-groups. Almost half of the sample (48%) was classified as low income and 41% had secondary school (or lower) as their highest level of education. Generally consistent with the national population distribution, just over half (54%) the sample resided in the eastern part of China versus 21% in central China and 25% in western China^32^.

Almost half of the respondents (47%) reported that they had never heard of e-cigarettes or vaping. There was some variation according to sex and age: 53% of females reported not having heard of e-cigarettes/vaping compared to 41% of males, and 45% of those aged 15–21 years had not heard of e-cigarettes/vaping compared to 50% of those aged 22–30 years. Twenty percent of the total sample reported being current or previous users of e-cigarettes (‘even once or twice’). Forty-two percent of the sample reported being a past or current user of tobacco cigarettes, and 6% described themselves as being current dual users of e-cigarettes and tobacco cigarettes. Males represented the majorities of those reporting previous (63%) or current (59%) e-cigarette use, being a current smoker (61%), and dual use (65%).

Among those previously or currently using e-cigarettes at least monthly (n=111), 30% (3% of the total sample) reported daily use (Table 1). The most popular forms of devices were those featuring replaceable screw-on cartridges (used by 63% of atleast-monthly users) and disposable versions (56%). The most frequently reported reasons for vaping were to reduce the number of cigarettes smoked (41%), because a friend uses them (39%), and to help quit smoking regular cigarettes (30%). E-cigarettes were most commonly obtained from vape shops (56%) and a friend aged ≥18 years (40%). The home was the location most often nominated as being where respondents used e-cigarettes (41%). The most preferred e-cigarette flavors were fruit (62%), mint (48%), and candy/chocolate/desserts (36%). There were no significant differences by sex, age, or smoking status on these variables.

**Table 1:** E-cigarette-related behaviors and motivations among previous and current at least monthly users by sex, age, and smoking status, among adolescents and young adults (aged 15–30 years) in China, November– December 2021 (N=111)

	*Total*	*Sex*		*Age (years)*		*Smoking status*		
		*Females*	*Males*	*15–21*	*22–30*	*Never*	*Previous*	*Current*
	*N=111 n (%)*	*N=47 n (%)*	*N=64 n (%)*	*N=57 n (%)*	*N=54 n (%)*	*N=2 n (%)*	*N=32 n (%)*	*N=77 n (%)*
**Daily use of e-cigarettes** (yes)	33 (30)	12 (26)	21 (33)	15 (26)	18 (33)	0 (0)	6 (19)	27 (35)
Device type*								
Replaceable screw-on cartridges	70 (63)	28 (60)	42 (66)	29 (51)	41 (76)	1 (50)	19 (59)	50 (65)
Disposable	62 (56)	28 (60)	34 (53)	29 (51)	33 (61)	1 (50)	14 (44)	47 (61)
Click-in pod	45 (41)	22 (47)	23 (36)	18 (32)	27 (50)	1 (50)	15 (47)	29 (38)
Refillable tank	40 (36)	20 (43)	20 (31)	21 (37)	19 (35)	1 (50)	12 (38)	27 (35)
Mod system	33 (30)	16 (34)	17 (27)	14 (25)	19 (35)	0 (0)	11 (34)	22 (29)
Most preferred flavors*								
Fruit	69 (62)	30 (64)	39 (61)	32 (56)	37 (69)	1 (50)	20 (63)	48 (62)
Mint	53 (48)	21 (45)	32 (50)	30 (53)	23 (43)	2 (100)	11 (34)	40 (52)
Candy, chocolate, desserts	40 (36)	18 (38)	22 (34)	26 (46)	14 (26)	0 (0)	12 (38)	28 (36)
Ice flavors	30 (27)	14 (30)	16 (25)	15 (26)	15 (28)	0 (0)	8 (25)	22 (29)
Tobacco	30 (27)	16 (34)	14 (22)	10 (18)	20 (37)	1 (50)	8 (25)	21 (27)
Menthol	29 (26)	15 (32)	14 (22)	10 (18)	19 (35)	0 (0)	7 (22)	22 (29)
**Reasons for vaping**								
To cut down on cigarettes smoked	46 (41)	14 (30)	32 (50)	24 (42)	22 (41)	NA	12 (38)	33 (43)
A friend used them	43 (39)	20 (43)	23 (36)	21 (37)	22 (41)	1 (50)	10 (31)	32 (42)
To help quit smoking regular cigarettes	33 (30)	12 (26)	21 (33)	12 (21)	21 (39)	NA	9 (28)	24 (31)
Out of curiosity	30 (27)	10 (21)	20 (31)	18 (32)	12 (22)	0 (0)	8 (25)	22 (29)
They taste better than regular cigarettes	19 (17)	8 (17)	11 (17)	10 (18)	9 (17)	0 (0)	7 (22)	12 (16)
They have appealing flavors	12 (11)	5 (11)	7 (11)	7 (12)	5 (9)	0 (0)	4 (13)	8 (10)
How/where obtained*								
Vape shop	62 (56)	28 (60)	34 (53)	27 (47)	35 (65)	1 (50)	19 (59)	42 (55)
Friend over 18 years old	44 (40)	20 (43)	24 (38)	22 (39)	22 (41)	1 (50)	13 (41)	30 (39)
Tobacconist/tobacco shop	28 (25)	13 (28)	15 (23)	15 (26)	13 (24)	2 (100)	5 (16)	21 (27)
Online	23 (21)	9 (19)	14 (22)	10 (18)	13 (24)	0 (0)	9 (28)	14 (18)
Where most used*								
At home, inside and outside	46 (41)	21 (45)	25 (39)	18 (32)	28 (52)	0 (0)	13 (41)	33 (43)
At other people’s homes	35 (32)	13 (28)	22 (34)	21 (37)	14 (26)	0 (0)	9 (28)	26 (34)
At parties	33 (30)	17 (36)	16 (25)	18 (32)	15 (28)	1 (50)	9 (28)	23 (30)
At home – outside	28 (25)	15 (32)	13 (20)	13 (23)	15 (28)	0 (0)	8 (25)	20 (26)
When drinking alcohol	19 (17)	6 (13)	13 (20)	6 (11)	13 (24)	2 (100)	4 (13)	13 (17)

* Selection of multiple response options permitted. No significant difference in all comparisons at p<0.001. NA: not applicable.

After excluding those indicating they had never heard of e-cigarettes/vaping, 46% of the remaining sample reported having at least one family member and 66% reported having at least one friend who used e-cigarettes (Table 2). There were some significant differences by sex, age, and smoking status. Compared to males and younger respondents, larger proportions of females and older respondents in this group reported having family members who vape. In addition, larger proportions of older respondents and previous/current smokers reported having friends who vape compared to younger and never-smoker respondents.

**Table 2:** External influences on using e-cigarettes by sex, age, and smoking status, among adolescents and young adults (aged 15–30 years) in China, November–December 2021 (N=560)

	*Total*	*Sex*		*Age (years)*		*Smoking status*			
		*Females*	*Males*	*15–21*	*22–30*	*Never*	*Previous*	*Current*	
*Total sample*	*N=560 n (%)*	*N=260 n (%)*	*N=300 n (%)*	*N=303 n (%)*	*N=257 n (%)*	*N=264 n (%)*	*N=107 n (%)*	*N=189 n (%)*	
**Number of family members using e-cigs**									
0	304 (54)	**116 (45)**	188 (63)***	**193 (64)**	111 (43)***	**165 (63)**	**38 (36)**	**101 (53)**	a
1	132 (24)	70 (27)	62 (21)	58 (19)	74 (29)	47 (18)	29 (27)	56 (30)	
2	86 (15)	52 (20)	34 (11)	38 (13)	48 (19)	39 (15)	28 (26)	19 (10)	
≥3	38 (7)	22 (8)	16 (5)	14 (5)	24 (9)	13 (5)	12 (11)	13 (7)	
**Number of close friends using e-cigs**									
0	192 (34)	84 (32)	108 (36)	**129 (43)**	63 (25)***	**132 (50)**	**18 (17)**	**42 (22)**	a,b
1	98 (18)	42 (16)	56 (19)	51 (17)	47 (18)	47 (18)	16 (15)	35 (19)	
2	104 (19)	54 (21)	50 (17)	44 (15)	60 (23)	37 (14)	27 (25)	40 (21)	
≥3	166 (30)	80 (31)	86 (29)	79 (26)	87 (34)	**48 (18)**	**46 (43)**	**72 (38)**	a,b
**Exposure to advertising** #									
Petrol station	248 (44)	98 (38)	150 (50)	146 (48)	102 (40)	**86 (33)**	**58 (54)**	**104 (55)**	a,b
Internet	218 (39)	94 (36)	124 (41)	113 (37)	105 (41)	**67 (25)**	**48 (45)**	**103 (55)**	a,b
TV, cinema, streaming services	179 (32)	79 (30)	100 (33)	102 (34)	77 (30)	**51 (19)**	**43 (40)**	**85 (45)**	a,b
Dummy variable results	343 (61)	142 (55)	201 (67)	190 (63)	153 (60)	**133 (50)**	**77 (72)**	**133 (70)**	a,b

E-cigs: e-cigarettes. Analyses exclude those who had never heard of e-cigarettes/vaping.

# Selected ‘Sometimes’ or ‘Often’ on 4-point scale: ‘Never’, ‘Rarely’, ‘Sometimes’, and ‘Often’. Bold text denotes significant differences.

*** Significant difference by sex/age at p<0.001.

a Never smoker significantly different to previous smoker at p<0.001.

b Never smoker significantly different to current smoker at p<0.001.

Almost half (44%) of those who were aware of e-cigarettes/vaping reported observing e-cigarette advertising at petrol stations and around one-third reported seeing such advertising on the Internet (39%) or TV/cinema/streaming services (32%). In total, 61% of this group had seen advertising in at least one of these formats. Reported exposure to these forms of advertising was significantly more prevalent among previous and current smokers compared to never smokers (Table 2).

Respondents’ beliefs about e-cigarettes are reported in Table 3. Almost three-quarters of respondents who were aware of e-cigarettes/vaping believed the products contain chemicals (71%) and are bad for health (71%). Two-thirds believed they contain nicotine (66%) and are addictive (66%). Around half believed they can explode and cause injury (53%) and that they can help people quit cigarettes (52%). However, 42% considered e-cigarette vapor to be harmless. There were few significant differences in beliefs among sub-groups, with a notable exception being a larger proportion of current smokers (62%) believing e-cigarettes can assist with quitting compared to previous (41%) smokers.

**Table 3:** E-cigarette-related beliefs and intentions by sex, age and smoking status, among adolescents and young adults (aged 15–30 years) in China, November–December 2021 (N=560)

	*Total*	*Sex*		*Age (years)*		*Smoking status*			
		*Females*	*Males*	*15–21*	*22–30*	*Never*	*Previous*	*Current*	
*Perceived e-cig characteristics#*	*N=560 n (%)*	*N=260 n (%)*	*N=300 n (%)*	*N=303 n (%)*	*N=257 n (%)*	*N=264 n (%)*	*N=107 n (%)*	*N=189 n (%)*	
Contain chemicals	396 (71)	175 (67)	221 (74)	220 (73)	176 (68)	174 (66)	71 (66)	151 (80)	
Are bad for your health	400 (71)	176 (68)	224 (75)	229 (76)	171 (67)	200 (76)	69 (64)	131 (69)	
Are addictive	368 (66)	169 (65)	199 (66)	213 (70)	155 (60)	185 (70)	68 (64)	115 (61)	
Contain nicotine	368 (66)	166 (64)	202 (67)	207 (68)	161 (63)	177 (67)	67 (63)	124 (66)	
Can explode and cause injury	296 (53)	139 (53)	157 (52)	**183 (60)**	113 (44)***	**154 (58)**	**38 (36)**	**104 (55)**	c
Help people quit cigarettes	289 (52)	132 (51)	157 (52)	160 (53)	129 (50)	**127 (48)**	**44 (41)**	**118 (62)**	b
Vapor is harmless water vapor	235 (42)	106 (41)	129 (43)	139 (46)	96 (37)	114 (43)	35 (33)	86 (46)	
**Never users’ intentions^**	N=346	N=177	N=169	N=199	N=147	N=255	N=37	N=54	
Curious to use	179 (52)	94 (53)	85 (50)	**80 (40)**	99 (67)***	**116 (45)**	**23 (62)**	**40 (74)**	a
Use if close friend offered	80 (23)	37 (21)	43 (25)	**31 (16)**	49 (33)***	**41 (16)**	**10 (27)**	**29 (54)**	a
Intend to use in next year	71 (21)	36 (20)	35 (21)	**27 (14)**	44 (30)***	**33 (13)**	**7 (19)**	**31 (57)**	a,b
**Minors** (aged 15–17 years)	N=86	N=21	N=65			N=62	N=10	N=14	
**Perceived ease of access in store**									
Not at all	28 (33)	7 (33)	21 (32)	NA	NA	26 (42)	1 (10)	1 (7)	
Somewhat	50 (58)	12 (57)	38 (58)	NA	NA	30 (48)	8 (80)	12 (86)	
Easy	8 (9)	2 (10)	6 (9)	NA	NA	6 (10)	1 (10)	1 (7)	
**Perceived ease of access online**									
Not at all	24 (28)	7 (33)	17 (26)	NA	NA	16 (26)	4 (40)	4 (29)	
Somewhat	41 (48)	8 (38)	33 (51)	NA	NA	35 (56)	2 (20)	4 (29)	
Easy	21 (24)	6 (29)	15 (23)	NA	NA	11 (18)	4 (40)	6 (43)	

E-cig: e-cigarette. Analyses exclude those who had never heard of e-cigarettes/vaping. Bold text denotes significant differences.

*** Significant difference by sex/age at p<0.001.

a Never vs current smoker significantly different at p<0.001.

b Previous vs current smoker significantly different at p<0.001.

c Never vs previous smoker significantly different at p<0.001.

# Selected ‘Agree’ or ‘Strongly Agree’ on 5-point scale: ‘Strongly Disagree’ to ‘Strongly Agree’.

^ Selected ‘Probably yes’ or ‘Definitely yes’ on a 4-point scale: ‘Definitely not’ to ‘Definitely yes’. NA: not applicable.

Among respondents who had heard of e-cigarettes but never used them, 52% were curious about vaping, 23% reported an intention to use e-cigarettes in the following year, and 21% said they would probably/definitely use an e-cigarette if offered one by a friend (Table 3). For all three susceptibility variables, proportions were significantly larger among older respondents and current smokers. Among minors who had heard of e-cigarettes, 67% considered it somewhat easy or easy to obtain e-cigarettes from stores and 72% considered it somewhat easy or easy to obtain e-cigarettes online (Table 3).

In the multivariable logistic regression model, current use of tobacco cigarettes (AOR=68.26; 95% C.I: 16.28–94.15, p<0.001) and previous (AOR=39.15; 95% CI: 28.69–162.42, p<0.001) use of tobacco cigarettes had the largest associations with e-cigarette ever use among those who had heard of e-cigarettes/vaping (Table 4). The number of friends who vape was also positively associated with ever use (AOR=1.76; 95% CI: 1.38–2.24, p<0.001), while a negative association was found for perceived addictiveness (AOR=0.47; 95% CI: 0.28–0.79, p<0.001). The other assessed independent variables (sex, age, education level, income, region of residence, perceived harmfulness, number of family members who vape, and exposure to advertising) were not found to be significantly associated with e-cigarette ever use in this sample.

**Table 4:** Logistic regression results: Factors associated with ever e-cigarette use among adolescents and young adults (aged 15–30 years) in China, November–December 2021 (N=506)^a^

*Factors*	*AOR*	*95% CI*	*Standard error*	*z*	p*
**Sex**					
Male (Ref.)	1				
Female	0.73	0.37–1.44	0.25	-0.91	0.36
**Age** (years)	0.96	0.88–1.06	0.05	-0.76	0.45
**Education level**					
Completed school (Ref.)	1				
Certificate/diploma	1.21	0.56–2.63	0.48	0.49	0.63
University qualification	1.67	0.75–3.73	0.68	1.24	0.21
**Income**					
Low (Ref.)	1				
Middle	0.82	0.33–2.04	0.38	-0.42	0.68
High	1.12	0.46–2.72	0.51	0.25	0.80
**Location**					
East (Ref.)	1				
Central	1.10	0.50–2.41	0.44	0.23	0.82
West	1.43	0.71–2.86	0.51	1.00	0.32
**Tobacco use status**					
Never used (Ref.)	1				
Previously users	39.15	16.28–94.15	17.53	8.19	**0.00**
Current users	68.26	28.69–162.42	30.19	9.55	**0.00**
Perceived addictiveness	0.47	0.28–0.79	0.12	-2.85	**0.00**
Perceived harmfulness	0.70	0.46–1.06	0.15	-1.68	0.09
Number of family member users	1.12	0.80–1.57	0.19	0.65	0.52
Number of friend users	1.76	1.38–2.24	0.22	4.60	**0.00**
Exposure to advertising	1.55	0.82–2.95	0.51	1.34	0.18

AOR: adjusted odds ratio.

a Excluding respondents who reported never having heard of e-cigarettes/vaping, those who reported ‘I don't know’ for the perceived harmfulness item, and those with missing data for the education and income items.

* Bold values significant at p<0.05.

Due to the provision of a description of e-cigarettes in the survey instrument, all respondents were asked about their support for various e-cigarette control policies. Majority support was found for 13 of the 14 assessed e-cigarette policies (Table 5). The exception was ‘E-cigarettes that do not contain nicotine should be prohibited’, for which only 46% of respondents selected ‘Agree’ or ‘Strongly agree’ on the five-point agreement scale. However, the means (calculated excluding ‘I don’t know’ responses) for all policies were above the neutral ‘3’ score (range: 3.39–4.20). Policies with at least three-quarters of respondents expressing agreement were: ‘E-cigarettes should be subject to the same regulations as tobacco cigarettes’, ‘There should be laws to prevent people aged <18 years from buying and using e-cigarettes’, ‘Nicotine concentration or volume in e-liquids should be at low levels that prevent users becoming more addicted’, ‘E-cigarette devices, e-liquids, and their packaging should have clearly visible health warning messages’, and ‘Advertising that is misleading, such as claiming e-cigarettes to be 'safe' or 'harmless', should be prohibited’.

**Table 5:** Support for e-cigarette policies, among adolescents and young adults (aged 15–30 years) in China, November–December 2021 (N=1062)

	*Agree^ n (%)*	*IDK n (%)*	*Mean*	*SD*
E-cigarettes should be subject to the same regulations as tobacco cigarettes	867 (82)	20 (2)	4.08	0.85
There should be laws to prevent people aged <18 years from buying and using e-cigarettes	854 (80)	41 (4)	4.20	0.86
Nicotine concentration or volume in e-liquids should be at low levels that prevent users becoming more addicted	804 (76)	53 (5)	4.10	0.87
E-cigarette devices, e-liquids, and their packaging should have clearly visible health warning messages	809 (76)	45 (4)	4.09	0.85
Advertising that is misleading, such as claiming e-cigarettes to be ‘safe’ or ‘harmless’, should be prohibited	800 (75)	47 (4)	4.10	0.91
E-cigarettes that contain nicotine should be prohibited	777 (73)	53 (5)	4.05	0.90
E-cigarette devices and liquids should be prohibited unless they have been proven to be safe and efficacious for smoking cessation	761 (72)	36 (3)	4.00	0.93
Advertisements with celebrities, cartoons, or other endorsements should be prohibited	752 (71)	54 (5)	3.99	0.99
E-cigarette advertising and promotion that may encourage use by young people should be prohibited	754 (71)	46 (4)	3.97	0.98
Flavors that may appeal to young people and contribute to addiction (e.g. confectionary, dessert, cannabis, soft drink, energy drink, fruit, and mint) should be prohibited	747 (70)	40 (4)	3.96	0.97
All e-cigarette flavors, including menthol, should be prohibited	662 (62)	44 (4)	3.73	1.10
E-cigarette packaging should be child-safe	588 (55)	40 (4)	3.42	1.41
E-cigarettes should be treated as if they are prescription medicines, i.e. only sold with a prescription in pharmacies	549 (52)	71 (7)	3.54	1.21
E-cigarettes that do not contain nicotine should be prohibited	485 (46)	96 (9)	3.39	1.22
Average	66	4	3.91	0.53

^ Selected agree or strongly agree on a 5-point ‘Strongly disagree’ to ‘Strongly agree’ scale. IDK: selected ‘I don't know’ response. Means calculated including all responses except ‘I don't know’. SD: standard deviation.

## DISCUSSION

In this sample of people aged 15–30 years in China, 47% reported being unaware of e-cigarettes/vaping. This was generally consistent with the results of the Global Adults Tobacco Survey China Project^9^, but substantially higher than previous research with middle and secondary school children where 70–74% were found to be aware of e-cigarettes^4,20^. This disparity is likely to be at least partially attributable to school-aged children being a small proportion of the total sample in the present study and awareness decreasing with age.

Twenty percent of the sample in the present study reported ever using e-cigarettes, 8% were current users, and 3% were daily users. The ever e-cigarette use rate found in this study is somewhat higher than reported in other studies of young adults in China in recent years despite consistent item wording^3,21^, which may be due to the different sampling frames but could indicate increasing vaping prevalence in this age group as per the findings of previous work^9^. The smoking rate of 27% for this sample is aligned with national prevalence data for people aged ≥15 years in China^2,33^.

As per previous research^5-7^, cessation- and social-related motivations dominated the reasons given for using e-cigarettes. While flavors were not a primary stated reason for vaping, fruit flavors were very popular, followed by mint and candy/chocolate/desserts. Most of those using e-cigarettes obtained them from vape shops and friends, and relatively few purchased them online. Likely reflecting the only recent introduction of bans on selling e-cigarettes to minors, majorities of those aged 15–17 years who were aware of e-cigarettes/vaping considered it somewhat easy or easy to source e-cigarettes in-store or online.

More than half of those who were aware of e-cigarettes/vaping but were never users indicated they were curious to use e-cigarettes, and more than one-fifth intended to use them in the next year or to use them if offered by a close friend. This indicates high levels of susceptibility among never users in this sample, despite the results relating to e-cigarette attitudes indicating that majorities believed that e-cigarettes contain chemicals, are bad for health, and are addictive. Of concern is that almost half of those who were aware of e-cigarettes/vaping considered e-cigarette vapor to be harmless. These findings support the repeated calls in the literature for greater public education on the nature and harms of e-cigarettes, with a particular focus on educating adolescents and young adults^3,6,10,13,21^.

Exposure to vaping-related stimuli was common among those aware of e-cigarettes/vaping. Almost two-thirds reported exposure to some form of e-cigarette advertising, and almost half had at least one family member and two-thirds had at least one friend who used e-cigarettes. The regression analyses showed having friends who use e-cigarettes to be significantly associated with ever use. Other significant predictors were current and previous smoking status and perceived addictiveness. Although in the frequency analyses males demonstrated higher previous and current e-cigarette use prevalence, gender was not a significant predictor of e-cigarette ever use in the regression model, indicating that other characteristics, in particular smoking status, are likely to be stronger influencing factors.

There was generally strong support for the assessed e-cigarette control policies. Many of these measures, in particular banning sales to youth, had been the subject of numerous calls for action^5,6,9^. Ten of the 14 policies received support from ≥70% of respondents, and all but one received majority support. The policy support outcomes are positive in light of the timing of data collection – the survey was administered just prior to the formal introduction of a range of restrictive measures. Overall, the results indicate the new regulations are likely to be met with widespread acceptance from members of this age group. A partial exception is suggested by the minority support observed for banning non-nicotine e-cigarettes. Given the demonstrated relationship in the health literature between policy support and compliance^34^, this element of the e-cigarettes regulatory framework may require particular monitoring and enforcement efforts.

### Limitations

The primary limitation of the present study was participant recruitment via a web panel provider, which may have resulted in a sample skewed on unassessed attributes (e.g. health literacy or sensation seeking tendencies). While this limits the generalizability of results, the application of demographic quotas provides some assurance of adequate coverage across the 15–30 years population subgroup. Although the sampling approach used in this study cannot be considered equivalent to periodic nationally representative data collection programs such as the Global Adults and Youth Tobacco Surveys, it provides supplementary data that are timely and cost-effective indicators of emerging trends in use of novel nicotine products. Second, a modest number of demographic characteristics was included in the analyses. Future research could incorporate additional variables, such as whether study participants reside in urban or rural areas. Third, the cross-sectional approach to data collection prevents any causal attributions, and as such the regression results can only be considered tentative. Fourth, while consistent with previous survey research in China^3,18^, the use of questionnaire items that asked respondents to report e-cigarette usage in terms of ‘even once or twice’ will have captured those who experimented but did not progress to regular use. Further research is needed to provide a more nuanced picture of youth vaping in China, especially now that restrictions are in place to reduce youth access.

## CONCLUSIONS

The results of the present study provide novel insights into young people’s attitudes and behaviors on the cusp of new production and marketing restrictions that seek to reduce e-cigarette harm, especially among young people. The timing of data collection and the broad range of factors assessed make the findings a baseline from which to measure changes in behavior resulting from regulatory reform. It appears that a sizeable minority of young people have been able to access e-cigarettes, with usage levels found to be highest among current and past smokers and those with friends who vape. Of interest will be changes in this situation once the new regulations have had time to take effect.

Overall, the results suggest that the substantial penetration of e-cigarettes into the youth market in China will make monitoring and enforcement of the new regulations critical for ensuring they meet the objective of minimizing vaping harm to young people. The strong link between smoking and vaping indicates the potential for exacerbated harms from dual use in this age group, highlighting the need to ensure young people are aware of the risks associated with dual use and ensuring effective smoking cessation assistance is made available. Concerns about e-cigarette addictiveness were negatively associated with vaping, indicating that this is an important area for public education to discourage uptake. The high levels of policy support observed among this age group are promising for compliance with the newly introduced suite of e-cigarette control regulations.

## Data Availability

The data supporting this research are available from the authors on reasonable request.
