# Supplementary material for: E-cigarette-related beliefs, behaviors, and policy support among young people in China
Source: Tob Induc Dis. 2023 Jan 23;21:09. doi: 10.18332/tid/156836 (PMC9869085; doi:10.18332/tid/156836)
Supplement: Supplementary file 1 [file TID-21-09-s1.pdf]

Supplementary materials

Supplementary Table S1: Response rate data (data collected November-December 2021 in China)

Respondent status	n
Starts^	1505
Completes	1062
Screeners	52
Quota Full	311
Disqualified (Quality / Panel Duplicates etc.)	2
Incompletes	78

^Individuals who elected to commence the survey that was promoted to them within the Pureprofile app via a generic ad that did not specify the survey topic.

Screened out respondents failed to meet the age eligibility criterion and disqualified respondents were those whose data was considered low quality (e.g., very short survey completion speed). Those classified as 'Incompletes' failed to complete the survey.

Supplementary Table S2: Sample profile by vaping and smoking status, China, 2021 (%)

	**Total**	**Never tried/used tobacco cigarettes or e-cigarettes**	**Previous e-cigarette user^**	**Current e-cigarette user**	**Previous smoker^**	**Current smoker**	**Current dual user**
	n = 1062	n = 603 (57%)	n = 127 (12%)	n = 87 (8%)	n = 166 (16%)	n = 284 (27%)	n = 65 (6%)
**Sex**							
Female	553 (52)	326 (54)	47 (37)	36 (41)	109 (66)	111 (39)	23 (35)
Male	509 (48)	277 (46)	80 (63)	51 (59)	57 (34)	173 (61)	42 (65)
**Age**							
15-17	148 (14)	122 (20)	12 (9)	8 (9)	10 (6)	15 (5)	6 (9)
18-20	326 (31)	212 (35)	37 (29)	30 (34)	36 (22)	76 (27)	25 (38)
21-23	179 (17)	115 (19)	26 (20)	8 (9)	22 (13)	42 (15)	5 (8)
24-26	152 (14)	78 (13)	23 (18)	8 (9)	23 (14)	47 (17)	5 (8)
27-30	257 (24)	76 (13)	29 (23)	33 (38)	75 (45)	104 (37)	24 (37)
**Location**							
East	578 (54)	327 (54)	70 (55)	56 (64)	83 (50)	165 (58)	44 (68)
Central	218 (21)	119 (20)	24 (19)	11 (13)	50 (30)	44 (15)	7 (11)
West	266 (25)	157 (26)	33 (26)	20 (23)	33 (20)	75 (26)	14 (22)
**Education**							
School	431 (41)	301 (50)	34 (27)	21 (24)	36 (22)	93 (33)	18 (28)
Diploma	385 (36)	230 (38)	47 (37)	18 (21)	57 (34)	94 (33)	13 (20)
University	244 (23)	71 (12)	46 (36)	48 (55)	72 (43)	97 (34)	34 (52)
Missing	2 (0)	1 (0)	0 (0)	0 (0)	1 (1)	0 (0)	0 (0)
**Income**							
Low	507 (48)	399 (66)	32 (25)	29 (33)	42 (25)	63 (22)	27 (42)
Medium	150 (14)	71 (12)	27 (21)	6 (7)	23 (14)	55 (19)	4 (6)
High	391 (37)	126 (21)	65 (51)	51 (59)	97 (58)	163 (57)	34 (52)
Missing	14 (1)	7 (1)	3 (2)	1 (1)	4 (2)	3 (1)	0 (0)

^Definitions of previous e-cigarette users and smokers include ‘even once or twice’ use.

© 2023 Pettigrew S. et al.
